# Research Progress on Strategies that can Enhance the Therapeutic Benefits of Mesenchymal Stromal Cells in Respiratory Diseases With a Specific Focus on Acute Respiratory Distress Syndrome and Other Inflammatory Lung Diseases

**DOI:** 10.3389/fphar.2021.647652

**Published:** 2021-04-19

**Authors:** Sara Rolandsson Enes, Anna D. Krasnodembskaya, Karen English, Claudia C. Dos Santos, Daniel J. Weiss

**Affiliations:** ^1^ Department of Experimental Medical Science, Faculty of Medicine, Lund University, Lund, Sweden; ^2^ Wellcome-Wolfson Institute for Experimental Medicine, School of Medicine, Dentistry, and Biomedical Sciences, Queens University, Belfast, United Kingdom; ^3^ Cellular Immunology Laboratory, Biology Department, Kathleen Lonsdale Institute for Human Health Research, Maynooth University, Maynooth, Ireland; ^4^ Interdepartmental Division of Critical Care, Department of Medicine and the Keenan Center for Biomedical Research, St. Michael’s Hospital, University of Toronto, Toronto, ON, Canada; ^5^ Department of Medicine, 226 Health Science Research Facility, Larner College of Medicine, University of Vermont, Burlington, VT, United States

**Keywords:** cell therapy, mesenchymal stromal cells (MSCs), mscs, lung diseases, respiratory diseases and disorders

## Abstract

Recent advances in cell based therapies for lung diseases and critical illnesses offer significant promise. Despite encouraging preclinical results, the translation of efficacy to the clinical settings have not been successful. One of the possible reasons for this is the lack of understanding of the complex interaction between mesenchymal stromal cells (MSCs) and the host environment. Other challenges for MSC cell therapies include cell sources, dosing, disease target, donor variability, and cell product manufacturing. Here we provide an overview on advances and current issues with a focus on MSC-based cell therapies for inflammatory acute respiratory distress syndrome varieties and other inflammatory lung diseases.

## Introduction

Severe respiratory diseases such as the acute respiratory distress syndrome (ARDS) and other inflammatory lung diseases remain a significant cause of mortality worldwide. For many of these end-stage diseases, there is no cure and current treatments aim to treat symptoms and/or delay disease progression. New treatment strategies are therefore critically needed. Mesenchymal stromal cells (MSCs) have been widely studied in the field of regenerative medicine for applications in the treatment of lung diseases and critical illnesses. MSCs are multipotent progenitor cells with the potential to secrete a spectrum of anti-inflammatory mediators, such as cytokines and extracellular vesicles (EVs). However, due to a lack of basic understanding of the *in vivo* modes of action and of scientific consensus concerning manufacturing, disease target, and study design the translation of efficacy from preclinical studies to the clinical settings have not yet been successful. Despite fundamental discoveries regarding the potential benefits of MSCs in pre-clinical models of lung diseases and other critical illnesses, more effort needs to be made to understand the *in vivo* fate of infused MSCs, particularly in clinical settings. Importantly, in addition to the MSC cell product, conditioned media and bioactive products such as EVs isolated from MSC cultures have been used in pre-clinical settings with promising results, however, these have been reviewed extensively in other publications (Abreu et al., 2016; Abreu et al., 2021) and will not be discussed further. Understanding how the diseased environment affects MSC therapeutic potential will make it possible to design more efficient clinical trials and to include patients that are more likely to respond to this type of treatment. This is particularly relevant for ARDS in which there may be different inflammatory phenotypes (Reilly et al., 2019). Here, we identify and summarize strategies to enhance the therapeutic potential of MSCs in respiratory diseases and provide a discussion regarding challenges in the manufacturing process and the importance of patient selection.

## Strategies to Enhance Therapeutic Potential of Mesenchymal Stromal Cells in Respiratory Diseases

Despite the enormous interest in using MSCs in clinical settings to treat respiratory lung diseases, the knowledge regarding the exact mechanism of action is limited. Today, the generally accepted hypothesis is that MSCs modulate the immune system through different paracrine effects (such as cytokines and extracellular vesicles), a hypothesis mainly based on pre-clinical studies [reviewed in (Fernanda Ferreira Cruz, 2019)].

### Inflammatory Environmental Effects on Therapeutic Behaviors

Preclinical evidence suggests that MSCs sense the environment through various damage-associated and pathogen-associated cell surface receptors and respond differentially depending on the environmental requirements (Traggiai et al., 2008; Romieu-Mourez et al., 2009; Waterman et al., 2010). For example, stimulation of Toll-like receptor 3 (TLR3) and Toll-like receptor 4 (TLR4) evoked very different MSC responses and appeared to polarize MSCs into two different immune regulatory phenotypes (Waterman et al., 2010). By exposing MSCs to LPS (a TLR4 agonist) the human MSCs differentiated toward a more pro-inflammatory phenotype with release of the cytokines IL-6 and IL-8. Poly (I:C) on the other hand (a TLR3 agonist), activated a more anti-inflammatory phenotype with release of mediators such as IDO, PGE2, and RANTES (Waterman et al., 2010). In addition, activation of MSC TLR4 signaling was demonstrated to be critical for MSC survival and therapeutic effect in a pre-clinical model of *E. coli*-induced acute lung injury, where MSCs isolated from TLR4 deficient mice had impaired survival under conditions of inflammatory stress *in vitro*, and were not therapeutically active *in vivo*. Mechanistically, it was shown that TLR4 pathway regulates signaling through PAR1 on MSCs and TLR4 stimulation leads to expression and secretion of prothrombin by MSCs (Gupta et al., 2018).

To understand the mechanisms by which MSCs act *in vivo*, Islam *et al.* induced ARDS by intratracheal installation of hydrochloric acid, mechanical ventilation, or a combination of the two methods (two-hit model). Following lung injury, MSCs were administered and the outcome was evaluated for each different lung injury model with relevant markers including Ashcroft lung injury score, pulmonary elastance, and lung collagen content (Islam et al., 2019). The authors found that the therapeutic effect of MSCs depended on the microenvironment at the time of administration. For example, MSCs administered into the mechanical ventilation induced ARDS were found to be protective. In contrast, no beneficial effect of MSC administration was noted following hydrochloric acid induced ARDS. To further investigate the microenvironment in the different models, a proteomic analysis of the BALF samples was performed. It was clearly demonstrated that the most enriched clusters of proteins upregulated in the hydrochloric acid model, in which the MSCs did not have a beneficial effect, consisted of proteins involved in inflammation, coagulation, and fibrosis. These proteomic data are somewhat surprising and contradictory since it would seem reasonable that injected MSCs would have effects on at least the inflammatory proteins.

In line with the findings by Islam *et al.*, human MSCs (hMSCs) exposed to clinical bronchoalveolar lavage fluid (BALF) samples obtained from patients with ARDS were less effective in promoting an anti-inflammatory monocyte phenotype than hMSCs exposed to BALF obtained from patients with other lung diseases including acute respiratory exacerbations of cystic fibrosis (CF) (Abreu et al., 2019a). Interestingly, neutralizing IL-6 resulted in promotion of the anti-inflammatory monocyte phenotype (Abreu et al., 2019b). This was a proof-of-concept study and the primary etiology on the ARDS patients used in these experiments were described as either pneumonia or other, and therefore it is not possible to compare these data with the results from the Islam *et al.* study. In another study, Morrison *et al.* exposed hMSCs to pooled samples from ARDS patients and found that ARDS-exposed MSCs promoted anti-inflammatory macrophage marker expression and increased the phagocytic capacity of human macrophages. The increased phagocytic capacity was partly attributed to the acquisition of the phagocytic receptor CD44 expressed in extracellular vesicles secreted by MSCs after exposure to ARDS BALF (Morrison et al., 2017). In similarity with the Abreu *et al.* study, the details of the etiology of ARDS were not well described, making it difficult to compare findings from the two studies. Moreover, the monocytes used in each study came from different species (murine RAW cells vs. human monocyte-derived macrophages), which might explain why different pathways were activated and thereby different outcomes were obtained. Another important difference is that the control group to which the results were compared to were very different, hMSCs exposed to BALF from other lung diseases vs. hMSCs exposed to healthy control BALF samples.

In parallel, hMSCs exposed *ex vivo* to BALF samples obtained from cystic fibrosis patients who had *Aspergillus* infection were rapidly killed. This effect on hMSCs was at least partly related to the mycotoxin, gliotoxin, produced by the fungus (Sara Rolandsson Enes, 2020). In line with these findings, exposure to serum from asthmatic mice resulted in increased levels of MSC cell death. Interestingly, in addition to increased cell death, exposure to serum also induced increased expression of anti-inflammatory mediators including IDO-1, TSG-6, IFN-γ, and IL-10. Administration of these pre-exposed MSCs into house dust mite (HDM)‐induced allergic asthma model demonstrated that serum-exposed MSCs improved lung mechanics to a greater extent that unexposed MSCs (Abreu et al., 2019a).

**FIGURE 1:**
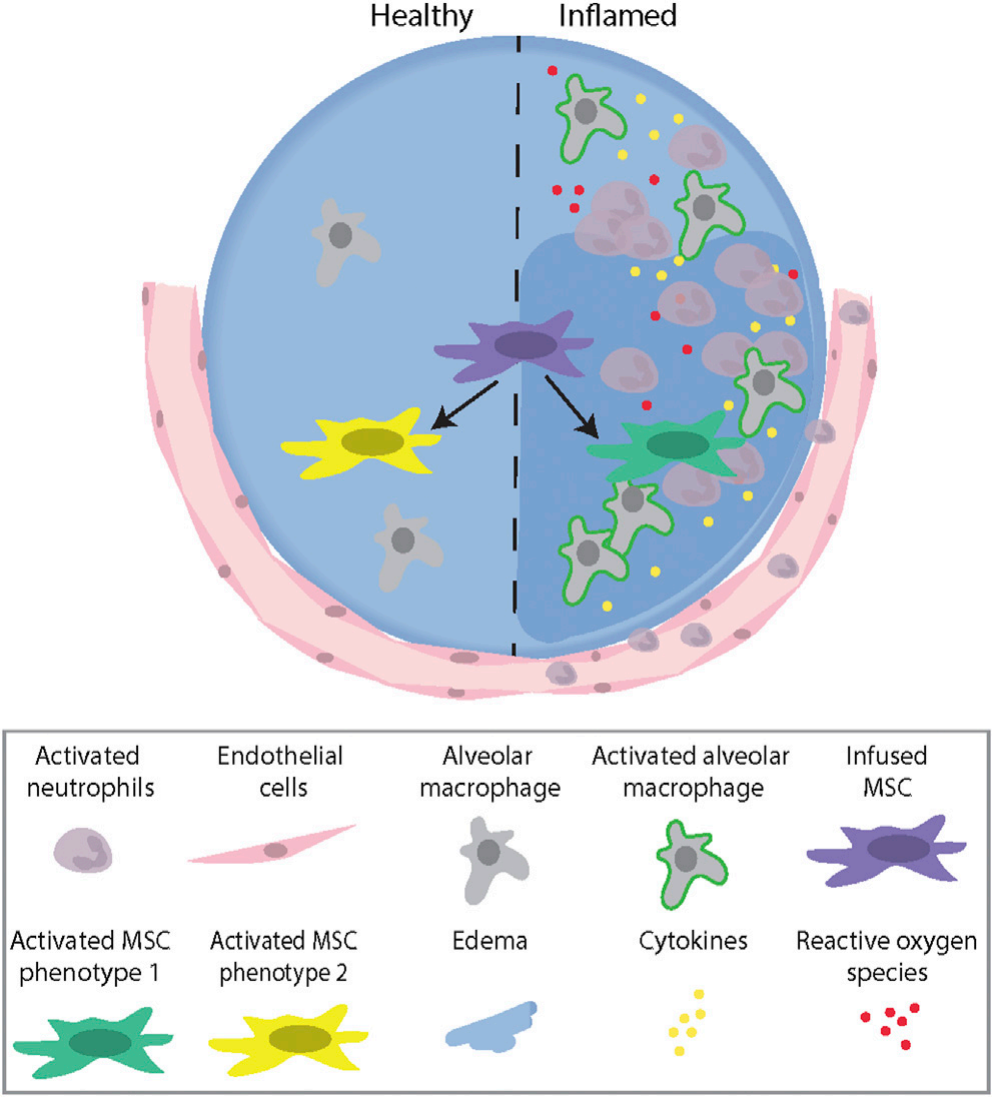
A schematic illustration describing the role of the microenvironment (healthy vs. inflamed) in activating MSCs to induce different therapeutic Phenotypes. Abbreviations: MSC, mesenchymal stromal cell.

The notion that the ability to “react” to MSCs is associated with their potential therapeutic benefit is supported by results from patients with graft-versus-host disease (GvHD) treated with MSCs. In these patients, only those who demonstrated high cytotoxic activity against the infused MSCs responded to the treatment. Using a murine GvHD model, it was demonstrated that recipient cytotoxic cells activated MSCs to undergo apoptosis through a perforin-dependent pathway. Perforin-induced MSC apoptosis was then further demonstrated to induce immunosuppression via indoleamine 2,3-diocygenase (IDO) production in recipient phagocytes (Galleu et al., 2017). This illustrates a growing appreciation that, whatever the MSCs themselves might do, the host responses to dead or dying MSCs can play a significant role in mitigating inflammation and injury (Weiss et al., 2019). Collectively, these studies demonstrate that an inflammatory microenvironment plays an important role in activating MSCs to induce different therapeutic and/or apoptotic phenotypes, which further highlight the need to understand the complex interaction between MSCs and the host environment **(** Figure 1).

### Pre-Activation of Mesenchymal Stromal Cells Prior to Administration

One way to take advantage of the current knowledge that MSCs are being activated by the inflammatory environment, is to pre-activate MSCs before administration. This could be done using different approaches; however, the most common method is to expose MSCs *ex vivo* to inflammatory cytokines such as IFN-γ and TNF-α (English et al., 2010). For example, MSCs exposed to IFN-γ had an increased immunosuppressive effect on T-lymphocytes. This suppressive effect was inhibited when an IFN-γ blocking antibody was added to the system (Krampera et al., 2006). Similarly, by using an IFN-γ knock out system it was demonstrated that endogenous IFN-γ was essential for MSC actions (Polchert et al., 2008). Functionally, pre-stimulation of hMSCs had superior protective effects in a humanized mouse model of acute GvHD (Tobin et al., 2013). In another study, MSCs stimulated with TNF-α demonstrated increased IL-6, IL-8, IL-2, and IFN-γ production compared to control MSCs. In this study, the investigators were also able to demonstrate that TNF-α was more important than IFN-γ regarding MSC secretion of IL-8 (Hemeda et al., 2010). By exposing MSCs to TNF-α in combination with IL-1β Murphey *et. al* observed that the immunomodulatory effects were enhanced compared to control cells (Murphy et al., 2019). In a recent study, it was demonstrated that TNF-α/IFN-γ/IL-1β exposed MSCs had a stronger IL-8 secretion than MSCs exposed to TNF-α/IFN-γ only. Moreover, this increased expression of IL-8 enhanced neutrophil recruitment, a recruitment that was suggested to be mediated trough activation of STAT5 and p38-MAPK signaling (Hackel et al., 2020). This is but one example of potential means of pre-activating MSCs.

Although hypoxia and high levels of oxidative stress are often seen in tissues with pathological inflammation and it is well known that oxygen levels can affect cell function (Cooper et al., 1958; Ivanovic et al., 2000; Choi et al., 2014; Choi et al., 2015), MSCs tend to be cultured at normal atmospheric oxygen levels. MSCs cultured at oxygen levels that better recapitulate the “destination” environment after administration significantly impacted their function. For example, MSCs cultured at low oxygen levels formed a greater number of colonies, had higher proliferation rates, and secreted higher levels of cytokines such as VEGF and FGF compared to MSCs cultured at 20% oxygen (Lennon et al., 2001; Choi et al., 2017; Kwon et al., 2017; Elabd et al., 2018). Moreover, MSCs co-cultured with bone marrow-derived lineage positive blood cells under hypoxic condition contributed to an increased proliferation and differentiation toward anti-inflammatory macrophages (Takizawa et al., 2017). More recently, the metabolic pathway used by MSCs has been shown to be important in determining immunomodulatory capacity (Liu et al., 2019). HIF-1α, the master hypoxia inducible transcription factor, regulated MSC immunomodulation and was associated with a metabolic switch from oxidative phosphorylation to glycolysis (Contreras-Lopez et al., 2020). These are all very interesting results, however, additional studies are needed in order to understand whether hypoxia activates an angiogenic phenotype that could be more appropriate for issue healing rather than immune modulatory. Altogether, modulating culture condition oxygen levels is an interesting approach to pre-activate MSCs, however there are still limited knowledge on this topic, particularly with respect to use of MSCs in respiratory diseases, and further studies are warranted.

Another physiological condition which frequently develops in chronic lung diseases or due to low tidal volume mechanical ventilation is hypercapnic acidosis. A study by Fergie *et al.* have demonstrated that culture of MSCs in 15% CO2 induced significant mitochondrial dysfunction and inhibited MSCs ability to promote reparative capacity of primary human pulmonary endothelial and distal lung epithelial cells (Fergie et al., 2019).

### Genetic Engineering of Mesenchymal Stromal Cells to Enhance Therapeutic Properties

Genetic engineering has been used to enhance MSC survival and therapeutic potential. Genetic engineering of MSCs has been used to induce expression of different cytokines, growth factors, transcription factors, miRNA, and enzymes (Damasceno et al., 2020). For example, overexpressing the pluripotent genes Oct4 and Sox2 in MSCs using liposomal transfection resulted in increased proliferation and differentiation capacity (Han et al., 2014). Moreover, rat bone marrow-derived MSCs transfected with the nerve growth factor receptor tropomyosin receptor kinase A (TrkA) showed increased survival and Schwann-like cell differentiation (Zheng et al., 2016), as well as improved nerve functional recovery and enhanced efficacy *in vivo* (Zheng et al., 2017). In a separate study, it was demonstrated that MSCs overexpressing insulin-like growth factor I (IGF-I) decreased collagen deposition in a liver fibrosis model compared to animals treated with GFP-MSCs or recombinant IGF-I (Fiore et al., 2015). Genetically engineered MSCs overexpressing anti-inflammatory cytokines such as IL-10 resulted in a reduction of pro-inflammatory cytokines such as IL-1β and TNF, and increased viability (Meng et al., 2018). Interestingly, MSCs overexpressing angiopoietin-1 were demonstrated to prevent LPS-induced acute lung injury and were more potent in decreasing inflammatory cells in bronchoalveolar lavage fluid (BALF) compared to untreated MSCs and saline treated groups. Also, levels of proinflammatory cytokines measured in BALF were further reduced in mice treated with angiopoietin-1 overexpressing MSCs compared to non-treated control MSCs (Mei et al., 2007).

### Mesenchymal Stromal Cells functional Heterogeneity and Tissue-specificity

Another important factor that likely plays a role in the absence of success of clinical trials is the lack of consistency between different studies such as use of MSCs isolated from different tissues and organs. Increasing evidence demonstrates that MSCs are tissue-specific cells that retain many tissue- and organ-specific functions and properties [reviewed in (Sara Rolandsson Enes, 2019)]. For example, MSCs isolated from bone marrow, skeletal muscle, periosteum, and perinatal cord blood differed in their transcriptomic signatures and *in vivo* differentiation profiles (Sacchetti et al., 2016). Moreover, MSCs isolated from lung tissue have also been demonstrated to differ from bone marrow-derived MSCs regarding proliferation rate, colony-forming potential, *in vivo* bone formation capacity, gene expression profile, secretome profile, and protein profile (Rolandsson et al., 2014; Rolandsson Enes et al., 2016; Rolandsson Enes et al., 2017). Although, tissue-specific properties have been observed by several investigators (Abreu et al., 2017), further parallel studies comparing MSCs from different sources need to be performed in order to roll out if one source is better than others to treat lung diseases. In addition to the MSC tissue-specificity, MSCs are also known to be a very heterogenous population, at least after standard *in vitro* tissue culture expansion. Whether it is possible to sort out specific MSC sub-populations with increased or specific therapeutic properties remains to be further explored.

## Strategies to Improve the Manufacturing Process and Clinical Outcome

Given the fairly high number of MSC-based clinical trials performed to date, it is surprising that there are no established consensus concerning MSC product manufacturing, dosing strategies, and which patient group or groups to target. It is well known that MSC properties change depending on the isolation process, number of passages, seeding density, culture surface, and if they have been cryopreserved or not. Regardless of this knowledge, a survey provided by the MSC committee of the International Society of Cell and Gene Therapy discovered that the current MSC manufacturing practice differs significant among different US academic centers (Phinney et al., 2019). Comparable lack of harmonization exists among MSC manufacturing facilities in Europe when a similar survey was performed (Trento et al., 2018). In this section, we will summarize and discuss some of the current strategies that could be used to improve the manufacturing process and clinical outcome.

### The Impact of Donor Variance on Mesenchymal Stromal Cells Properties

In addition to the above mentioned challenges such as source, seeding density, and cryopreservation, it is important to acknowledge the impact of donor variance on MSC numbers, but more importantly, on the MSC therapeutic potential. It is well known that MSC properties are affected by factors such as donor age, gender, and harvest site (Zaim et al., 2012; Martin et al., 2016; Rolandsson Enes et al., 2016; Mehrian et al., 2020). For example, MSCs isolated from donors at different ages will have different proliferation rates and *in vitro* differentiation potentials (Zaim et al., 2012; Mehrian et al., 2020). Therefore, it is important to take this into account when manufacturing MSCs for both basic studies and clinical trials and to find strategies that facilitate easy identification of clinically potent MSCs. One such strategy could potentially be to maximize the number of batches that one can obtain from a good donor. However, it is important to remember that MSCs manufactured in large quantities can become ineffective or altered, even from that single donor. When finally obtaining expanded MSCs of desired qualities from a good donor, it is important to pay attention to how the cells are being stored and finally administered to patients. In the report from the MSC pre-conference organized by the International Society of Cell Therapy (ISCT) held in 2018, the difference in potency between cryopreserved MSCs given as a “bedside thaw” formulation and cultured MSCs was highlighted (Nolta et al., 2020). Further, it has also been demonstrated by Francois *et. al.* that cryopreserved MSCs need a recovery phase before infused to patients in order to retain their immunomodulatory activity *in vitro* (Francois et al., 2012). However, interestingly recent data by Tan *et al.* demonstrated that there was comparable immunomodulatory efficacy of fresh and thawed MSCs (Tan et al., 2019).

### Predicting the Best Suited Mesenchymal Stromal Cells Cell Product Using Machine Learning

Artificial intelligence, including machine learning, is an interesting area of research which has been given a lot of attention during the last years. Machine learning algorithms and other artificial intelligence approaches may be trained to recognize underlaying relationships in a dataset and thereby perform tasks such as classification, recognition, and counting of lung nodules on chest CT to predict cancer risk and navigate clinical decision options (Khemasuwan et al., 2020). This approach has also been tested in an attempt to characterize MSCs as a step forward toward improved MSC manufacturing process and a better MSC cell product. In a recent study, machine learning was used to profile MSCs based on cell morphology. Here, images of MSCs at low and high passages from 10 different donors exposed to different doses of IFN-γ (0, 10, and 50 ng/ml) were taken and analyzed using visual stochastic neighbor embedding (viSNE) technique. Several morphological sub-populations of MSCs were found after IFN-γ exposure, and it was further suggested that this model could be used to predict immunosuppressive capacity of different MSC lots (Marklein et al., 2019). In another study, machine learning was used to predict MSC population doubling time using *ex vivo* expanded MSCs from multiple donors at different ages and passages. Data from 131 donors from all ages were used to build a predictive model for cultured MSC population doubling time in passage 1 to 4. As suggested by the authors, this model can be used as a tool during the manufacturing process in order to predict the time it takes to reach a desired number of MSCs from a specific donor (Mehrian et al., 2020). This is a new and very exciting area, which hopefully can lead to improvements, standardization, and simplifications in the MSC manufacturing process.

### The Importance of Predicting Clinical Responders

In addition to optimizing MSC manufacturing and potential pre-conditioning, it is equally important to predict patients that are more likely to respond to this type of treatment. In previous clinical studies, MSCs have been administered to patients with a variety of lung diseases including both chronic and acute diseases (Tzouvelekis et al., 2013; Weiss et al., 2013; Chambers et al., 2014; Zheng et al., 2014; Wilson et al., 2015; Glassberg et al., 2017). However, based on the general hypothesis that MSCs mainly act via secreted factors in combination with the fact that MSCs have been demonstrated to be cleared from the lungs within a few days, makes it unlikely that MSC-based therapy can remodel destroyed tissue found in patients with end-stage chronic lung diseases (Armitage et al., 2018; Rolandsson Enes and Weiss, 2020). It is more likely that patients with lung diseases involving acute inflammatory response and/or infection such as sepsis/septic shock and ARDS will respond better to this type of treatment. Even if preferentially well suited for the treatment of acute lung injury, patient heterogeneity as in the case of ARDS is critical and identifying those individuals or sub-populations that are more likely to benefit from MSC treatment is critical. For example, in a multicenter phase II clinical trial using MSCs to treat graft-versus-host disease (GvHD) it was demonstrated that a sub-population of MSC-treated patients had a complete response, and those patients further had an increased survival rate compared to partial-responders and non-responders (Le Blanc et al., 2008). Similarly, in a retrospectively study of children suffering from GvHD that were treated with MSCs, it was observed that complete responders had an increased survival rate compared to partial-responders and non-responders (Ball et al., 2013). Therefore, it would be very interesting, and important, to identify biological factors that differentiate the complete responders from the other two groups. While there may be different strategies to accomplish this, Martin *et al.* makes a compelling argument on investing more resources on mechanistical studies and the design of future clinical trials based on molecular markers found in subpopulations of patients (Martin et al., 2019).

### The Importance of Large-Scale Cell Manufacturing and Successful Cell Delivery

For clinical translation several challenges need to be addressed including how to generate the high number of good quality MSCs needed for large human trials, as well as how to produce and store MSCs for future clinical treatments. For example, ARDS is an acute severe disease with a very rapid onset and there is insufficient time to start cultivating MSCs when the patient is admitted to hospital thus the cell product needs to be a cryopreserved ready-to-use product. Furthermore, isolation processes, passage number, density strategies, growth media and cultivation properties (e.g., plastic vs 3D environments) each can influence the biological properties of MSCs (reviewed in (Sara Rolandsson Enes DJW and Burgess IHH, 2019)) are necessary to ensure therapeutic efficient MSCs. Another challenge is that the lung is a complex organ, and it is critical to find the most optimal delivery route where MSCs reach all compartments in the lung. In preclinical studies of lung diseases, the most common delivery route is still intravenous administration, however for lung diseases there are a number of examples in which intratracheal administration can be as or even more effective (Sally Yunsun Kim WC and Burgess IHH, 2019). For example, using a naphthalene-induced airway injury model Wong *et al.* demonstrated that a larger number of epithelial cells reached the lung when they were administered through transtracheal delivery over intravenous cell delivery (Wong et al., 2007). It is however important to remember that some lung diseases, such as COPD and ARDS, are systemic diseases and therefore different delivery routes might be good for some but not others.

### Challenges Related to Conducting Clinical Trials During the COVID-19 Outbreak

The global coronavirus pandemic has increased the number of initiated MSC clinical trials dramatically, and there are a number of challenges related to the conducting trials such as having a good cell product, as discussed above, enrolling patients, assessing outcomes, and ethical issues. For example, a global pandemic aim to find a rapid and effective treatment however it is important that this occurs in a transparent manner following appropriate ethical guidelines (Khoury et al., 2020; Khoury et al., 2021). As discussed by Khoury *et al.* it is also very important to include recognized end-point such as length of ICU, ventilator-free days, and overall mortality. Another important issue to considerate, especially for treatment of COVID-19 induced ARDS, is when to enroll patients and when to initiate MSC treatment, but also which COVID-19 patient population to target (Khoury et al., 2020). The issues discussed in this section is highly relevant for the ongoing COVID-19 MSC clinical trials, however they are equally important to consider in all cell-based therapies for pulmonary diseases.

## Summary and Final Remarks

The enthusiasm for using MSC-based therapy to treat severe lung disorders is great and it is currently increasing due to the COVID-19 pandemic. Unfortunately, the very promising pre-clinical studies performed in animal models of lung diseases has not yet translated into improved clinical outcomes in clinical trials. The field is still struggling with the lack of knowledge regarding the fate of administered MSCs, lack of standardized protocols for manufacturing of MSCs, and how to determine which patients are more likely to respond to this type of treatment. Despite these limitations, there are several interesting studies demonstrating that MSCs can be activated and modified in order to enhance their therapeutic efficacy using either genetic modifications or environmental factors including inflammatory cytokines and hypoxia. Moreover, recent studies have used artificial intelligence as a tool to predict which MSCs are better to be used in clinical trials, a strategy that would improve the manufacturing process substantially and hopefully lead to a better and more effective MSC cell product. We can conclude that currently there is no clear path to reconcile the issues discussed in this review, however one potential solution might be that investigators begin to combine all aspects of potential benefits into a unified effort to establish a greater search for where benefit could occur.

## References

[B1] Abreu S. C. Weiss D. J. Rocco P. R. (2016). Extracellular vesicles derived from mesenchymal stromal cells: a therapeutic option in respiratory diseases? Stem Cel Res Ther 7 (1), 53. 10.1186/s13287-016-0317-0 PMC4831172 27075363

[B2] Abreu S. C. Antunes M. A. Xisto D. G. Cruz F. F. Branco V. C. Bandeira E. (2017). Bone marrow, adipose, and lung tissue-derived murine mesenchymal stromal cells release different mediators and differentially affect airway and lung parenchyma in experimental asthma. Stem Cell Transl Med 6 (6), 1557–1567. 10.1002/sctm.16-0398 PMC5689762 28425576

[B3] Abreu S. C. Xisto D. G. de Oliveira T. B. Blanco N. G. de Castro L. L. Kitoko J. Z. (2019a). Serum from asthmatic mice potentiates the therapeutic effects of mesenchymal stromal cells in experimental allergic asthma. STEM CELLS Translational Med. 8 (3), 301–312. 10.1002/sctm.18-0056 PMC6392406 30426724

[B4] Abreu S. C. Enes S. R. Dearborn J. Goodwin M. Coffey A. Borg Z. D. (2019b). Lung inflammatory environments differentially alter mesenchymal stromal cell behavior. Am J Physiol Lung Cell Mol Physiol. 317 (6), L823-L831. 10.1152/ajplung.00263.2019 31553626 PMC6962599

[B5] Abreu S. C. Lopes-Pacheco M. Weiss D. J. Rocco P. R. M. (2021). Mesenchymal stromal cell-derived extracellular vesicles in lung diseases: current status and perspectives. Front Cel Dev Biol 9, 600711. 10.3389/fcell.2021.600711 PMC7917181 33659247

[B6] Armitage J. Tan D. B. A. Troedson R. Young P. Lam K. V. Shaw K. (2018). Mesenchymal stromal cell infusion modulates systemic immunological responses in stable COPD patients: a phase I pilot study. Eur. Respir. J. 51 (3). 10.1183/13993003.02369-2017 29348155

[B7] Ball L. M. Bernardo M. E. Roelofs H. van Tol M. J. Contoli B. Zwaginga J. J. (2013). Multiple infusions of mesenchymal stromal cells induce sustained remission in children with steroid-refractory, grade III-IV acute graft-versus-host disease. Br. J. Haematol. 163 (4), 501–509. 10.1111/bjh.12545 23992039

[B8] Chambers D. C. Enever D. Ilic N. Sparks L. Whitelaw K. Ayres J. (2014). A phase 1b study of placenta-derived mesenchymal stromal cells in patients with idiopathic pulmonary fibrosis. Respirology. 19 (7), 1013–1018. 10.1111/resp.12343 25039426

[B9] Choi J. R. Pingguan-Murphy B. Wan Abas W. A. B. Noor Azmi M. A. Omar S. Z. Chua K. H. (2014). Impact of low oxygen tension on stemness, proliferation and differentiation potential of human adipose-derived stem cells. Biochem. Biophysical Res. Commun. 448 (2), 218–224. 10.1016/j.bbrc.2014.04.096 24785372

[B10] Choi J. R. Pingguan-Murphy B. Wan Abas W. A. Yong K. W. Poon C. T. Noor Azmi M. A. (2015). *In situ* normoxia enhances survival and proliferation rate of human adipose tissue-derived stromal cells without increasing the risk of tumourigenesis. PLoS One. 10 (1), e0115034. 10.1371/journal.pone.0115034 25615717 PMC4304807

[B11] Choi J. R. Yong K. W. Wan Safwani W. K. Z. (2017). Effect of hypoxia on human adipose-derived mesenchymal stem cells and its potential clinical applications. Cell. Mol. Life Sci. 74 (14), 2587–2600. 10.1007/s00018-017-2484-2 28224204 PMC11107561

[B12] Contreras Lopez R. Elizondo-Vega R. Paredes M. J. Luque-Campos N. Torres M. J. Tejedor G. (2020). HIF1*α*-dependent metabolic reprogramming governs mesenchymal stem/stromal cell immunoregulatory functions. FASEB J. 34 (6), 8250–8264. 10.1096/fj.201902232R 32333618

[B13] Cooper P. D. Burt A. M. Wilson J. N. (1958). Critical effect of oxygen tension on rate of growth of animal cells in continuous suspended culture. Nature. 182 (4648), 1508–1509. 10.1038/1821508b0 13613312

[B14] Damasceno P. K. F. de Santana T. A. Santos G. C. Orge I. D. Silva D. N. Albuquerque J. F. (2020). Genetic engineering as a strategy to improve the therapeutic efficacy of mesenchymal stem/stromal cells in regenerative medicine. Front. Cel Dev Biol. 8, 737. 10.3389/fcell.2020.00737 PMC7471932 32974331

[B15] Elabd C. Ichim T. E. Miller K. Anneling A. Grinstein V. Vargas V. (2018). Comparing atmospheric and hypoxic cultured mesenchymal stem cell transcriptome: implication for stem cell therapies targeting intervertebral discs. J. Transl Med. 16 (1), 222. 10.1186/s12967-018-1601-9 30097061 PMC6086019

[B16] English K. French A. Wood K. J. (2010). Mesenchymal stromal cells: facilitators of successful transplantation? Cell Stem Cell. 7 (4), 431–442. 10.1016/j.stem.2010.09.009 20887949

[B17] Fergie N. Todd N. McClements L. McAuley D. O'Kane C. Krasnodembskaya A. (2019). Hypercapnic acidosis induces mitochondrial dysfunction and impairs the ability of mesenchymal stem cells to promote distal lung epithelial repair. FASEB j. 33 (4), 5585–5598. 10.1096/fj.201802056r 30649987 PMC6436662

[B18] Fernanda Ferreira Cruz P. R. M. R. (2019). in: Burgess IHH Janette K. ed. Stem Cell-Based Therapy for Lung Disease. Cham, Switzerland: Springer.

[B19] Fiore E. J. Bayo J. M. Garcia M. G. Malvicini M. Lloyd R. Piccioni F. (2015). Mesenchymal stromal cells engineered to produce IGF-I by recombinant adenovirus ameliorate liver fibrosis in mice. Stem Cell Development 24 (6), 791–801. 10.1089/scd.2014.0174 PMC4356192 25315017

[B20] Francois M. Copland I. B. Yuan S. Romieu-Mourez R. Waller E. K. Galipeau J. (2012). Cryopreserved mesenchymal stromal cells display impaired immunosuppressive properties as a result of heat-shock response and impaired interferon-gamma licensing. Cytotherapy 14 (2), 147–152. 10.3109/14653249.2011.623691 22029655 PMC3279133

[B21] Galleu A. Riffo-Vasquez Y. Trento C. Lomas C. Dolcetti L. Cheung T. S. (2017). Apoptosis in mesenchymal stromal cells induces *in vivo* recipient-mediated immunomodulation. Sci. Transl Med. 9 (416). 10.1126/scitranslmed.aam7828 29141887

[B22] Glassberg M. K. Minkiewicz J. Toonkel R. L. Simonet E. S. Rubio G. A. DiFede D. (2017). Allogeneic human mesenchymal stem cells in patients with idiopathic pulmonary fibrosis via intravenous delivery (aether): a phase I safety clinical trial. Chest. 151 (5), 971–981. 10.1016/j.chest.2016.10.061 27890713 PMC6026255

[B23] Gupta N. Sinha R. Krasnodembskaya A. Xu X. Nizet V. Matthay M. A. (2018). The TLR4-PAR1 *Axis* regulates bone marrow mesenchymal stromal cell survival and therapeutic capacity in experimental bacterial pneumonia. Stem Cells. 36 (5), 796–806. 10.1002/stem.2796 29396891 PMC5918231

[B24] Hackel A. Aksamit A. Bruderek K. Lang S. Brandau S. (2020). TNF-alpha and IL-1beta sensitize human MSC for IFN-gamma signaling and enhance neutrophil recruitment. Eur. J. Immunol. 51 (2), 319-330. 10.1002/eji.201948336 32845509

[B25] Han S.-M. Han S.-H. Coh Y.-R. Jang G. Chan Ra J. Kang S.-K. (2014). Enhanced proliferation and differentiation of Oct4- and Sox2-overexpressing human adipose tissue mesenchymal stem cells. Exp. Mol. Med. 46, e101. 10.1038/emm.2014.28 24946789 PMC4081551

[B26] Hemeda H. Jakob M. Ludwig A.-K. Giebel B. Lang S. Brandau S. (2010). Interferon-*γ* and tumor necrosis factor-*α* differentially affect cytokine expression and migration properties of mesenchymal stem cells. Stem Cell Development 19 (5), 693–706. 10.1089/scd.2009.0365 20067407

[B27] Islam D. Huang Y. Fanelli V. Delsedime L. Wu S. Khang J. (2019). Identification and modulation of microenvironment is crucial for effective mesenchymal stromal cell therapy in acute lung injury. Am. J. Respir. Crit. Care Med. 199 (10), 1214–1224. 10.1164/rccm.201802-0356oc 30521764

[B28] Ivanovic Z. Sbarba P. D. Trimoreau F. Faucher J.-L. Praloran V. (2000). Primitive human HPCs are better maintained and expanded *in vitro* at 1 percent oxygen than at 20 percent. Transfusion. 40 (12), 1482–1488. 10.1046/j.1537-2995.2000.40121482.x 11134568

[B29] Khemasuwan D. Sorensen J. S. Colt H. G. (2020). Artificial intelligence in pulmonary medicine: computer vision, predictive model and COVID-19. Eur. Respir. Rev. 29 (157), 200181. 10.1183/16000617.0181-2020 33004526 PMC7537944

[B30] Khoury M. Cuenca J. Cruz F. F. Figueroa F. E. Rocco P. R. M. Weiss D. J. (2020). Current status of cell-based therapies for respiratory virus infections: applicability to COVID-19. Eur. Respir. J. 55 (6), 2000858. 10.1183/13993003.00858-2020 32265310 PMC7144273

[B31] Khoury M. Ikonomou L. Dominici M. LeBlanc K. Levine B. L. Weiss D. J. (2021). The coronavirus pandemic: a pitfall or a fast track for validating cell therapy products? Stem Cell Dev 30 (3), 119–127. 10.1089/scd.2020.0122 33307968

[B32] Krampera M. Cosmi L. Angeli R. Pasini A. Liotta F. Andreini A. (2006). Role for interferon-*γ* in the immunomodulatory activity of human bone marrow mesenchymal stem cells. Stem Cells. 24 (2), 386–398. 10.1634/stemcells.2005-0008 16123384

[B33] Kwon S. Y. Chun S. Y. Ha Y.-S. Kim D. H. Kim J. Song P. H. (2017). Hypoxia enhances cell properties of human mesenchymal stem cells. Tissue Eng. Regen. Med. 14 (5), 595–604. 10.1007/s13770-017-0068-8 30603513 PMC6171625

[B34] Le Blanc K. Frassoni F. Ball L. Locatelli F. Roelofs H. Lewis I. (2008). Mesenchymal stem cells for treatment of steroid-resistant, severe, acute graft-versus-host disease: a phase II study. Lancet. 371 (9624), 1579–1586. 10.1016/S0140-6736(08)60690-X 18468541

[B35] Lennon D. P. Edmison J. M. Caplan A. I. (2001). Cultivation of rat marrow-derived mesenchymal stem cells in reduced oxygen tension: effects on *in vitro* and *in vivo* osteochondrogenesis. J. Cel. Physiol. 187 (3), 345–355. 10.1002/jcp.1081 11319758

[B36] Liu Y. Yuan X. Muñoz N. Logan T. M. Ma T. (2019). Commitment to aerobic glycolysis sustains immunosuppression of human mesenchymal stem cells. STEM CELLS Translational Med. 8 (1), 93–106. 10.1002/sctm.18-0070 PMC6312448 30272389

[B37] Marklein R. A. Klinker M. W. Drake K. A. Polikowsky H. G. Lessey-Morillon E. C. Bauer S. R. (2019). Morphological profiling using machine learning reveals emergent subpopulations of interferon-gamma-stimulated mesenchymal stromal cells that predict immunosuppression. Cytotherapy. 21 (1), 17–31. 10.1016/j.jcyt.2018.10.008 30503100

[B38] Martin I. De Boer J. Sensebe L. Therapy MSCCotISfC (2016). A relativity concept in mesenchymal stromal cell manufacturing. Cytotherapy. 18 (5), 613–620. 10.1016/j.jcyt.2016.02.004 27059199

[B39] Martin I. Galipeau J. Kessler C. Le Blanc K. Dazzi F. (2019). Challenges for mesenchymal stromal cell therapies. Sci. Transl Med. 11 (480), eaat2189. 10.1126/scitranslmed.aat2189 30787168

[B40] Mehrian M. Lambrechts T. Marechal M. Luyten F. P. Papantoniou I. Geris L. (2020). Predicting *in vitro* human mesenchymal stromal cell expansion based on individual donor characteristics using machine learning. Cytotherapy. 22 (2), 82–90. 10.1016/j.jcyt.2019.12.006 31987754

[B41] Mei S. H. J. McCarter S. D. Deng Y. Parker C. H. Liles W. C. Stewart D. J. (2007). Prevention of LPS-induced acute lung injury in mice by mesenchymal stem cells overexpressing angiopoietin 1. Plos Med. 4 (9), e269. 10.1371/journal.pmed.0040269 17803352 PMC1961632

[B42] Meng X. Li J. Yu M. Yang J. Zheng M. Zhang J. (2018). Transplantation of mesenchymal stem cells overexpressing IL10 attenuates cardiac impairments in rats with myocardial infarction. J. Cel Physiol. 233 (1), 587–595. 10.1002/jcp.25919 28322445

[B43] Morrison T. J. Jackson M. V. Cunningham E. K. Kissenpfennig A. McAuley D. F. O’Kane C. M. (2017). Mesenchymal stromal cells modulate macrophages in clinically relevant lung injury models by extracellular vesicle mitochondrial transfer. Am. J. Respir. Crit. Care Med. 196 (10), 1275–1286. 10.1164/rccm.201701-0170oc 28598224 PMC5694830

[B44] Murphy N. Treacy O. Lynch K. Morcos M. Lohan P. Howard L. (2019). TNF‐*α*/IL‐1*β*-licensed mesenchymal stromal cells promote corneal allograft survival via myeloid cell‐mediated induction of Foxp3 + regulatory T cells in the lung. FASEB j. 33 (8), 9404–9421. 10.1096/fj.201900047r 31108041

[B45] Nolta J. A. Galipeau J. Phinney D. G. (2020). Improving mesenchymal stem/stromal cell potency and survival: proceedings from the International Society of Cell Therapy (ISCT) MSC preconference held in May 2018, Palais des Congres de Montreal, Organized by the ISCT MSC Scientific Committee. Cytotherapy. 22 (3), 123–126. 10.1016/j.jcyt.2020.01.004 32067856

[B46] Phinney D. G. Galipeau J. Gene T. Msc Committee Of The International Society Of C (2019). Manufacturing mesenchymal stromal cells for clinical applications: a survey of Good Manufacturing Practices at U.S. academic centers. Cytotherapy. 21 (7), 782–792. 10.1016/j.jcyt.2019.04.003 31182333

[B47] Polchert D. Sobinsky J. Douglas G. Kidd M. Moadsiri A. Reina E. (2008). IFN-*γ* activation of mesenchymal stem cells for treatment and prevention of graft versus host disease. Eur. J. Immunol. 38 (6), 1745–1755. 10.1002/eji.200738129 18493986 PMC3021120

[B48] Reilly J. Calfee C. Christie J. (2019). Acute respiratory distress syndrome phenotypes. Semin. Respir. Crit. Care Med. 40 (1), 019–030. 10.1055/s-0039-1684049 PMC6657496 31060085

[B49] Rolandsson Enes S. Ahrman E. Palani A. Hallgren O. Bjermer L. Malmstrom A. (2017). Quantitative proteomic characterization of lung-MSC and bone marrow-MSC using DIA-mass spectrometry. Sci. Rep. 7 (1), 9316. 10.1038/s41598-017-09127-y 28839187 PMC5570998

[B50] Rolandsson Enes S. Andersson Sjoland A. Skog I. Hansson L. Larsson H. Le Blanc K. (2016). MSC from fetal and adult lungs possess lung-specific properties compared to bone marrow-derived MSC. Sci. Rep. 6, 29160. 10.1038/srep29160 27381039 PMC4933903

[B51] Rolandsson Enes S. Weiss D. J. (2020). Cell therapy for lung disease: current status and future prospects. Curr. Stem Cel Rep 6, 30–39. 10.1007/s40778-020-00171-5

[B52] Rolandsson S. Andersson Sjoland A. Brune J. C. Li H. Kassem M. Mertens F. (2014). Primary mesenchymal stem cells in human transplanted lungs are CD90/CD105 perivascularly located tissue-resident cells. BMJ Open Respir. Res. 1 (1), e000027. 10.1136/bmjresp-2014-000027 PMC4212711 25478178

[B53] Romieu-Mourez R. François M. Boivin M.-N. Bouchentouf M. Spaner D. E. Galipeau J. (2009). Cytokine modulation of TLR expression and activation in mesenchymal stromal cells leads to a proinflammatory phenotype. J. Immunol. 182 (12), 7963–7973. 10.4049/jimmunol.0803864 19494321

[B54] Sacchetti B. Funari A. Remoli C. Giannicola G. Kogler G. Liedtke S. (2016). No identical “mesenchymal stem cells” at different times and sites: human committed progenitors of distinct origin and differentiation potential are incorporated as adventitial cells in microvessels. Stem Cel Rep. 6 (6), 897–913. 10.1016/j.stemcr.2016.05.011 PMC4912436 27304917

[B55] Sara Rolandsson Enes GW-T (2019). In: Burgess IHH Janette K. ed. Stem Cell-Based Therapy for Lung Disease. Cham, Switzerland: Springer.

[B56] Soraia C. Abreu T. H. H. Hoffman Evan. Jacob Dearborn. Ashare Alix. Singh Sidhu Karatatiwant. Sara Rolandsson Enes (2020). Differential effects of the cystic fibrosis lung inflammatory environment on mesenchymal stromal cells. Am J Physiol Lung Cell Mol Physiol*.* 319 (6), L908-L925. 10.1152/ajplung.00218.2020 32901521 PMC7792680

[B57] Sara Rolandsson Enes DJW (2019). In: Burgess IHH Janette K. ed. Stem Cell-Based Therapy for Lung Disease. Cham, Switzerland: Springer.

[B58] Sally Yunsun Kim WC (2019). In: Burgess IHH Janette K. ed. Stem Cell-Based Therapy for Lung Disease. Cham, Switzerland: Springer.

[B59] Takizawa N. Okubo N. Kamo M. Chosa N. Mikami T. Suzuki K. (2017). Bone marrow-derived mesenchymal stem cells propagate immunosuppressive/anti-inflammatory macrophages in cell-to-cell contact-independent and -dependent manners under hypoxic culture. Exp. Cel Res. 358 (2), 411–420. 10.1016/j.yexcr.2017.07.014 28712928

[B60] Tan Y. Salkhordeh M. Wang J. P. McRae A. Souza-Moreira L. McIntyre L. (2019). Thawed mesenchymal stem cell product shows comparable immunomodulatory potency to cultured cells in vitro and in polymicrobial septic animals. Sci. Rep. 9 (1), 18078. 10.1038/s41598-019-54462-x 31792313 PMC6889371

[B61] Tobin L. M. Healy M. E. English K. Mahon B. P. (2013). Human mesenchymal stem cells suppress donor CD4+T cell proliferation and reduce pathology in a humanized mouse model of acute graft-versus-host disease. Clin. Exp. Immunol. 172 (2), 333–348. 10.1111/cei.12056 23574329 PMC3628335

[B62] Traggiai E. Volpi S. Schena F. Gattorno M. Ferlito F. Moretta L. (2008). Bone marrow-derived mesenchymal stem cells induce both polyclonal expansion and differentiation of B cells isolated from healthy donors and systemic lupus erythematosus patients. Stem Cells. 26 (2), 562–569. 10.1634/stemcells.2007-0528 18024418

[B63] Trento C. Bernardo M. E. Nagler A. Kuci S. Bornhauser M. Kohl U. (2018). Manufacturing mesenchymal stromal cells for the treatment of graft-versus-host disease: a survey among centers affiliated with the European society for blood and marrow transplantation. Biol. Blood Marrow Transpl. 24 (11), 2365–2370. 10.1016/j.bbmt.2018.07.015 PMC6299357 30031938

[B64] Tzouvelekis A. Paspaliaris V. Koliakos G. Ntolios P. Bouros E. Oikonomou A. (2013). A prospective, non-randomized, no placebo-controlled, phase Ib clinical trial to study the safety of the adipose derived stromal cells-stromal vascular fraction in idiopathic pulmonary fibrosis. J. Transl Med. 11, 171. 10.1186/1479-5876-11-171 23855653 PMC3722100

[B65] Waterman R. S. Tomchuck S. L. Henkle S. L. Betancourt A. M. (2010). A new mesenchymal stem cell (MSC) paradigm: polarization into a pro-inflammatory MSC1 or an Immunosuppressive MSC2 phenotype. PLoS One. 5 (4), e10088. 10.1371/journal.pone.0010088 20436665 PMC2859930

[B66] Weiss D. J. Casaburi R. Flannery R. LeRoux-Williams M. Tashkin D. P. (2013). A placebo-controlled, randomized trial of mesenchymal stem cells in COPD. Chest. 143 (6), 1590–1598. 10.1378/chest.12-2094 23172272 PMC4694112

[B67] Weiss D. J. English K. Krasnodembskaya A. Isaza-Correa J. M. Hawthorne I. J. Mahon B. P. (2019). The necrobiology of mesenchymal stromal cells affects therapeutic efficacy. Front. Immunol. 10, 1228. 10.3389/fimmu.2019.01228 31214185 PMC6557974

[B68] Wilson J. G. Liu K. D. Zhuo H. Caballero L. McMillan M. Fang X. (2015). Mesenchymal stem (stromal) cells for treatment of ARDS: a phase 1 clinical trial. Lancet Respir. Med. 3 (1), 24–32. 10.1016/S2213-2600(14)70291-7 25529339 PMC4297579

[B69] Wong A. P. Dutly A. E. Sacher A. Lee H. Hwang D. M. Liu M. (2007). Targeted cell replacement with bone marrow cells for airway epithelial regeneration. Am. J. Physiol. Lung Cel Mol Physiol. 293 (3), L740–L752. 10.1152/ajplung.00050.2007 17616650

[B70] Zaim M. Karaman S. Cetin G. Isik S. (2012). Donor age and long-term culture affect differentiation and proliferation of human bone marrow mesenchymal stem cells. Ann. Hematol. 91 (8), 1175–1186. 10.1007/s00277-012-1438-x 22395436

[B71] Zheng G. Huang L. Tong H. Shu Q. Hu Y. Ge M. (2014). Treatment of acute respiratory distress syndrome with allogeneic adipose-derived mesenchymal stem cells: a randomized, placebo-controlled pilot study. Respir. Res. 15, 39. 10.1186/1465-9921-15-39 24708472 PMC3994204

[B72] Zheng M. Duan J. He Z. Wang Z. Mu S. Zeng Z. (2017). Transplantation of bone marrow stromal stem cells overexpressing tropomyosin receptor kinase A for peripheral nerve repair. Cytotherapy. 19 (8), 916–926. 10.1016/j.jcyt.2017.04.007 28571657

[B73] Zheng M. Duan J. He Z. Wang Z. Mu S. Zeng Z. (2016). Overexpression of tropomyosin receptor kinase A improves the survival and Schwann-like cell differentiation of bone marrow stromal cells in nerve grafts for bridging rat sciatic nerve defects. Cytotherapy. 18 (10), 1256–1269. 10.1016/j.jcyt.2016.06.015 27497699

